# Sorting of Early and Late Flagellar Subunits After Docking at the Membrane ATPase of the Type III Export Pathway

**DOI:** 10.1016/j.jmb.2007.09.080

**Published:** 2007-12-07

**Authors:** Graham P. Stafford, Lewis D.B. Evans, Rita Krumscheid, Paraminder Dhillon, Gillian M. Fraser, Colin Hughes

**Affiliations:** Cambridge University Department of Pathology, Tennis Court Road, Cambridge CB2 1QP, UK

**Keywords:** flagella assembly, type III export, sorting mechanism, export chaperone

## Abstract

The bacterial flagellum assembles in a strict order, with structural subunits delivered to the growing flagellum by a type III export pathway. Early rod-and-hook subunits are exported before completion of the hook, at which point a subunit-specificity switch allows export of late filament subunits. This implies that in bacteria with multiple flagella at different stages of assembly, each export pathway can discriminate and sort unchaperoned early and chaperoned late subunits. To establish whether subunit sorting is distinct from subunit transition from the cytosol to the membrane, in particular docking at the membrane-associated FliI ATPase, the pathway was manipulated *in vivo*. When ATP hydrolysis by the FliI ATPase was disabled and when the pathway was locked into an early export state, both unchaperoned early and chaperoned late subunits stalled and accumulated at the inner membrane. Furthermore, a chaperone that attenuates late subunit export by stalling when docked at the wild-type ATPase also stalled at the ATPase in an early-locked pathway and inhibited export of early subunits in both native and early-locked pathways. These data indicate that the pathways for early and late subunits converge at the FliI ATPase, independent of ATP hydrolysis, before a distinct, separable sorting step. To ascertain the likely signals for sorting, the export of recombinant subunits was assayed. Late filament subunits unable to bind their chaperones were still sorted accurately, but chaperoned late subunits were directed through an early-locked pathway when fused to early subunit N-terminal export signal regions. Furthermore, while an early subunit signal directed export of a heterologous type III export substrate through both native and early-locked pathways, a late subunit signal only directed export via native pathways. These data suggest that subunits are distinguished not by late chaperones but by N-terminal export signals of the subunits themselves.

Bacterial motility is commonly conferred by cell surface flagella, comprising a long helical filament that is connected by a flexible hook to a central rod in the cell envelope basal body that also houses the flagellar motor.^1–3^ Flagella substructures are assembled in strict sequence, with formation of the basal body and rod structures preceding polymerisation of the hook and, finally, the filament subunits.^1–3^ The order of assembly is achieved by sequential expression of the gene hierarchy^4^^,^^5^ and by a subunit-specificity switch in the flagellar type III export pathway. This ensures that, prior to hook completion, only ‘early’ rod-and-hook subunits are exported,^6^^,^^7^ while those forming the later distal substructures of the filament, filament cap and hook–filament junction are not. The outline of the subunit-specificity switching mechanism is evident. When the hook reaches its mature length, a signal is transmitted by the hook length control protein FliK to the integral membrane export component FlhB, triggering a switch in subunit specificity to allow export of late subunits.^6–9^ Nevertheless, peritrichously flagellated bacteria such as *Escherichia coli* and *Salmonella* have multiple flagella at different stages of assembly,^10^ so an individual export pathway potentially encounters both unchaperoned early and chaperoned late subunits from the cytosol. This implies that early and late subunits are discriminated and sorted by the pathway.

We have previously shown that late filament subunits are piloted by their chaperones to dock at the membrane-associated FliI ATPase.^11^ Here we manipulate the export pathway to determine whether subunit docking and sorting are separable and sequential events. We also assess the relative influence of subunit export signals and bound export chaperones in discriminating early and late subunits.

## Stalling of early and late subunits at the membrane in an early-locked pathway attenuated in ATP hydrolysis

To examine the relationship between the proposed sorting step and subunit transition from the cytosol to the inner membrane, we aimed to generate stalled export intermediates of both early and late subunits. Our previous work had exploited export-defective chaperones to stall late cognate (hook–filament junction) subunits, which they piloted to and docked at the membrane-associated FliI ATPase.^11^ To similarly interrupt the movement of unchaperoned early subunits, we attenuated FliI ATP hydrolysis, which, as in other export systems,^12^^,^^13^ is envisaged to drive unfolding and export of substrates engaged at the membrane machinery,^14^ in this case prior to assembly into the growing flagellum. After creating single-amino-acid substitutions in the active site region, one variant was chosen for full study, variant FliI_E211A_, which is mutated immediately adjacent to the Walker A motif. ATP was still bound by FliI_E211A_ [*K*_m_ = 0.2 mM, compared to wild type (1 mM); triplicate assays ± 15%] but was poorly hydrolysed [*V*_max_ = 0.22 μmol min^− 1^mg^− 1^ compared to wild type (2.30)] in a coupled assay in the presence of phospholipids.^15^

**Fig. 1:**
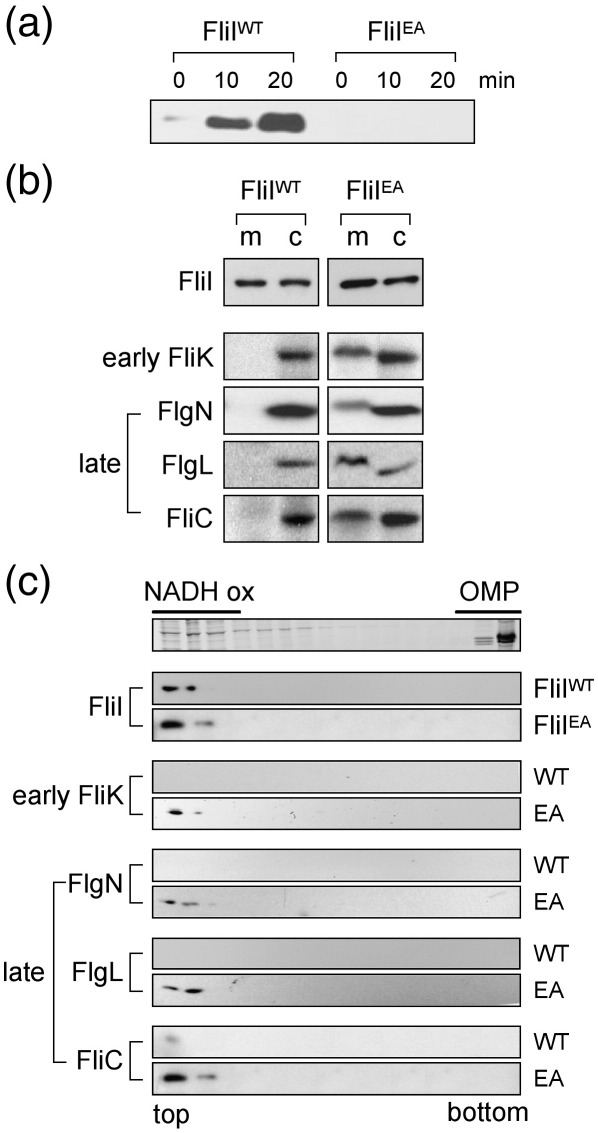
Membrane accumulation of early and late subunits in the pathway attenuated by enzymatically impaired FliI ATPase. (a) FliC export, assayed by immunoblotting, filtered supernatants from midexponential Luria broth (LB) cultures (*A*_600_ = 1.0) of Δ*fliIflgKflgM* cells (made by P22 transduction combined with the method of Datsenko and Wanner^27^) expressing in *trans* either wild-type FliI (FliI^WT^) or variant FliI_E211A_ (FliI^EA^) from pBAD33 (0.1% arabinose). A Δ*fliIflgKflgM* strain containing empty pBAD33 was shown to be nonmotile and attenuated in the export of early FliK subunit and late FliC subunit (data not shown). (b) *Salmonella fliIflgKflgM* cultures expressing wild-type FliI^WT^ or variant FliI^EA^ separated into membrane (m) and cytoplasmic (c) fractions.^11^^,^^16^ Immunoblotted for FliI ATPase, FlgN chaperone and subunits. (c) Separation of the membrane fractions into outer membrane (OMP; Coomassie stained) and inner membrane (NADH oxidase marker) by sucrose gradient ultracentrifugation (0.8–2.0 M^11^^,^^16^ top and bottom of the gradient indicated). Proteins immunoblotted using antisera described above.

Export supported by FliI_E211A_ was assayed in a *fliIflgKflgM* triple mutant (by our previously published method^11^). The resulting pathway is not subject to negative feedback (via the FlgM anti-sigma factor) arising from the disabling of the export apparatus (*fliI* ATPase), and late and early subunits are thus constitutively synthesized. When wild-type *fliI* is expressed in *trans*, exported late subunits such as filament subunit FliC accumulate in the culture supernatant as a result of the *flgK* hook–junction lesion that precludes filament polymerisation (Fig. 1a). Export of FliC was severely attenuated by substitution of FliI by FliI_E211A_. Like the wild-type ATPase, FliI_E211A_ assembled into hexamers *in vitro* in the presence of phospholipids and the short-arm crosslinker disuccinimidylglutamate (Supplementary Data), and cell fractionation and sucrose gradient ultracentrifugation^11^^,^^16^ showed that, *in vivo*, it localised normally to the inner membrane (Fig. 1b and c).

The *fliIflgKflgM* pathway containing the nonhydrolysing FliI_E211A_ is locked into an early export state.^6,7,11^ *In vivo* localisation of nonexported subunits in this pathway revealed (Fig. 1b and c) that the unchaperoned early subunit FliK^17^ accumulated as a membrane-associated intermediate in a FliI-dependent manner. This indicates that, like chaperoned late subunits, unchaperoned early subunits can be stalled at the membrane, putatively docked at the FliI ATPase. If late subunits are sorted before they dock at FliI, then late subunit–chaperone complexes should not accumulate at the membrane in the FliI_E211A_ early-locked *fliIflgKflgM* pathway, but they should accumulate if sorting occurs after late subunit docking. The *in vivo* fractionation and sucrose gradients of *fliIflgKflgM* cells expressing FliI_E211A_ (Fig. 1b and c) revealed that the late subunits FliC and FlgL and the FlgN chaperone^18^^,^^19^ accumulate, like early FliK, at the inner membrane.

The data indicate that FliI enzymatic activity is not required for *in vivo* docking of late subunits at the membrane ATPase (compatible with *in vitro* interaction of virulence chaperones with a catalytically inactive type III export ATPase^14^), and they indicate that this is also true for unchaperoned early subunits. Furthermore, they argue that sorting is separable from docking at FliI, occurring most likely afterwards, and that progression to sorting requires ATP hydrolysis by FliI.

## Early and late subunits converge at the ATPase prior to sorting

We have described a late FlgN chaperone variant (now called FlgN^rel^, as it putatively fails to release from the ATPase) that attenuates export of cognate and noncognate late subunits when expressed in *trans* in wild-type pathways, with chaperoned subunits trapped after docking at the membrane FliI, accumulating chaperone–subunit–ATPase intermediates.^11^ We used this dominant-negative chaperone variant to extend indications that chaperoned late subunits engage the wild-type FliI ATPase before sorting, asking whether FlgN^rel^-stalled membrane intermediates accumulate in an early export-locked pathway and attenuate early subunit export.

**Fig. 2:**
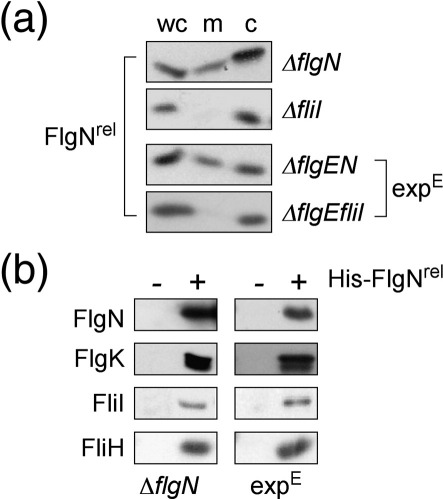
Membrane accumulation of early and late subunits in an early-locked pathway attenuated by stalling docked chaperone FlgN^rel^. (a) Localisation of FlgN^rel^ (expressed in *trans* by 0.01% arabinose) in the whole cell (wc), membrane (m) and cytoplasm (c)^11^^,^^15^ of the Δ*flgN* and Δ*fliI* pathways, and in the isogenic (exp^E^) early-locked pathways Δ*flgEN* and Δ*flgEfliI*. (b) Affinity copurification of stalled docking complexes by His–FlgN^rel^ bait (+)^11^ from native Δ*flgN* and early-locked (exp^E^; Δ*flgEN*) pathways [(−) vector-only controls]. Cell extracts were incubated with Ni–NTA resin [20 mM tris(hydroxymethyl)aminomethane–HCl pH 8.0, 300 mM NaCl and 5 mM imidazole] before washing (10 mM imidazole) and elution in SDS sample buffer. FlgN chaperone, FlgK cognate subunit and ATPase complex components FliI and FliH were detected by immunoblotting.

Cell fractionation was performed to establish FlgN^rel^ localisation and putative interaction with the ATPase in the actively secreting early-locked pathway lacking the hook protein FlgE and the wild-type FlgN chaperone (exp^E^; Δ*flgE*Δ*flgN*), and also in native pathways of Δ*flgN* control (exp^E + L^) that export early subunits and then late subunits after completion of the hook substructure. The results (Fig. 2a) show that FlgN^rel^ localised to the membrane in a FliI-dependent manner in both pathways. When His-FlgN^rel^ was used as bait in *in vivo* affinity chromatography (Fig. 2b), cognate subunit FlgK, FliI and its regulator FliH were all copurified, showing that ATPase–chaperone–subunit intermediate complexes were formed in early-locked pathways analogous to native pathways.

**Fig. 3:**
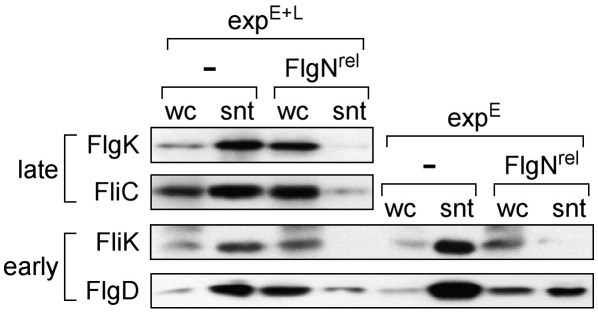
Attenuation of early and late subunits export by stalling FlgN^rel^. Export of subunits by native Δ*fliD* (exp^E + L^) and early-locked Δ*flgE* (exp^E^) pathways containing FlgN^rel^ [expressed using 0.01% arabinose; (−) vector-only controls], assayed following precipitation from supernatants (snt) of midexponential LB cultures (wc, whole culture) by SDS-PAGE and immunoblotting for early (FliK and FlgD) and late (FliC and FlgK) subunits.

These findings support the earlier indication that both unchaperoned early and chaperoned late subunits engage the export ATPase before they are sorted for export or exclusion, and furthermore suggest that the export pathways for the two classes of subunit converge at the ATPase. To substantiate the idea of convergence, we again used the docked but stalled late FlgN^rel^ to assess whether it could attenuate export of not only chaperoned late subunits but also early subunits in native export pathways of Δ*flgN*. The results (Fig. 3) confirm that export of cognate (FlgK) and noncognate (FliC) chaperoned late subunits is reduced by 5- to 10-fold and reveal a comparable attenuation in the export of unchaperoned early subunits FlgD and FliK. Significantly, they show that FlgN^rel^ caused a comparable reduction of FlgD and FliK export in an early export-locked (Δ*flgE*Δ*flgN*; exp^E^) pathway (Fig. 3). These FlgN^rel^ experiments strengthen the indication from those using FliI_E211A_, that is, that the pathways for unchaperoned early and chaperoned late subunits converge at the ATPase before progressing (dependent on ATP hydrolysis) to sorting.

## Are subunits discriminated by their own signals or by late chaperones?

**Fig. 4:**
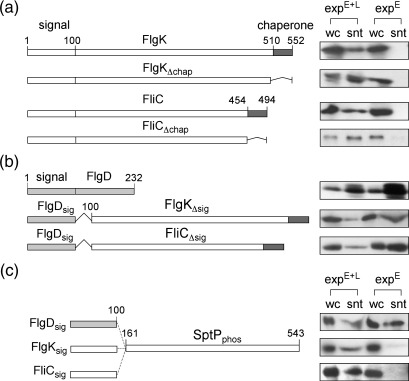
Influence of subunit domains on sorting. (a) Export of chaperoned late subunits (FlgK and FliC) and their variants that cannot bind chaperone (FlgK_Δchap_ and FliC_Δchap_) in export pathways that are either early locked (exp^E^; Δ*flgEKL*^27^ Δ*flgE*, SJW1353 acquired from Ohnishi *et al.*^28^) or native (exp^E + L^; Δ*flgKL* or Δ*fliC*). Proteins from whole cells (wc) and supernatants (snt) were immunoblotted with FlgK or FliC antisera. (b) Export of FlgD and recombinant late subunits FlgK_Δsig_ and FliC_Δsig_ lacking amino acids 1–100 fused to residues 1–100 of early FlgD in export pathways (described above) that are either early locked (exp^E^) or native (exp^E + L^). Proteins from whole cells (wc) and supernatants (snt) were immunoblotted with FlgD, FlgK or FliC antisera. (c) Export of recombinant fusion proteins comprising putative early or late subunit N-terminal signal regions (FlgD_sig_, FlgK_sig_ and FliC_sig_; amino acids 1–100) fused to the signal-less SptP tyrosine phosphatase domain (SptP_phos_; residues 161–543) in early locked (exp^E^; Δ*flgE*) and native (exp^E + L^; Δ*fliC*) pathways. Proteins were immunoblotted with SptP antisera (V. Koronakis, University of Cambridge). Genes encoding variant wild-type and variant FliC, FlgK, FlgD and SptP were amplified by overlap extension PCR using *Salmonella* chromosomal DNA as template. PCR products were inserted into XbaI–HindIII restriction sites of the pBAD18 expression vector. Recombinant genes were expressed (LB, 0.01% arabinose) and proteins were assayed as in Fig. 2b. Control experiments performed in isogenic Δ*fliI* and Δ*flgEfliI* strains (created by P22 transduction of Δ*fliI* allele into Δ*flgE*) showed that none of the recombinant proteins was exported (data not shown).

An obvious difference between early and late subunits is that only late subunits are bound by export chaperones, acting as cytosolic bodyguards and pilots for docking at the membrane export ATPase.^11^^,^^20^^,^^21^ Chaperones could act as flagellar sorting signals, labelling subunits for rejection during the early stages of flagella assembly. This is especially so as in a virulence type III secretion system, bound chaperones (e.g., *Salmonella* InvB chaperone of the SopE effector), are reported to form part of the secretion signal, preventing promiscuous export through the flagellar pathway.^20,21^ If this were true, late subunits from which C-terminal polymerisation and chaperone-binding domains are deleted might be exported as early subunits. To test this possibility, we assessed the export of recombinant FliC and FlgK late subunit variants lacking their chaperone-binding domains (FlgK_Δchap_, and FliC_Δchap_)^6^^,^^18^^,^^22^ and found (Fig. 4a) that, although these variants were exported in native pathways that export both early and late subunits (exp^E + L^), neither variant was exported in an early-locked pathway (exp^E^; i.e., the unchaperoned subunits were still faithfully sorted by the export pathway). We then assessed the export of recombinant subunits in which the N-terminal export signal residues 1–100 (FlgD_sig_) were fused to truncated late FliC (FliC_Δsig_) or FlgK (FlgK_Δsig_) lacking their N-terminal export signals^23^ but still able to bind their respective chaperones. Like wild-type FlgD, these hybrid subunits were exported by both early export-locked (exp^E^) and native export (exp^E + L^) pathways (Fig. 4b), substantiating the view that chaperones are not a sorting signal to preclude late subunit export before hook completion. The marginal (twofold) reduction in the export of early subunits (FlgD, FlgD_sig_–FliC_Δsig_, FlgD_sig_–FlgK_Δsig_) in native pathways (Fig. 4b; exp^E + L^), compared to the early export-locked strain (exp^E^), possibly indicates that once an export apparatus has switched specificity, it no longer accepts early subunits for export (a view compatible with observations in *Yersinia* T3SS indicating that once pathways have switched specificity to late effectors, they do not export early substrates^24^).

These assays also suggest that the subunit N-terminal 100 residues contain both export and sorting signals. To confirm this, we constructed recombinant subunits comprising the N-terminal 100 residues of the late subunit FliC (FliC_sig_) or FlgK (FlgK_sig_), or the early subunit FlgD (FlgD_sig_) fused to the catalytic phosphatase domain of the *Salmonella* SPI-1 SptP effector (residues 161–543; SptP_phos_)^14^ and assayed export in early export-locked and wild-type equivalent pathways. As expected, FlgD_sig_–SptP_phos_ fusion was exported in both early-locked (exp^E^) and wild-type (exp^L + E^) pathways (Fig. 4c). In contrast, the FliC_sig_–SptP_phos_ and FlgK_sig_–SptP_phos_ fusion proteins were exported only in the wild-type pathway (exp^E + L^) and not by the early export-locked pathway (exp^E^) (Fig. 4c). This supports our view that the N-terminal regions contain sufficient information to determine sorting as a late subunit. While there is primary sequence similarity among the N-terminal regions of rod subunits, there seems to be little identity between these and other early subunits^25^^,^^26^ and no obvious identity distinguishing the N-terminal regions of late proteins. This suggests that subunit sorting might rely on the recognition of structural features specific to each subunit class.

## Figures and Tables

**Supplementary Fig. 1:**
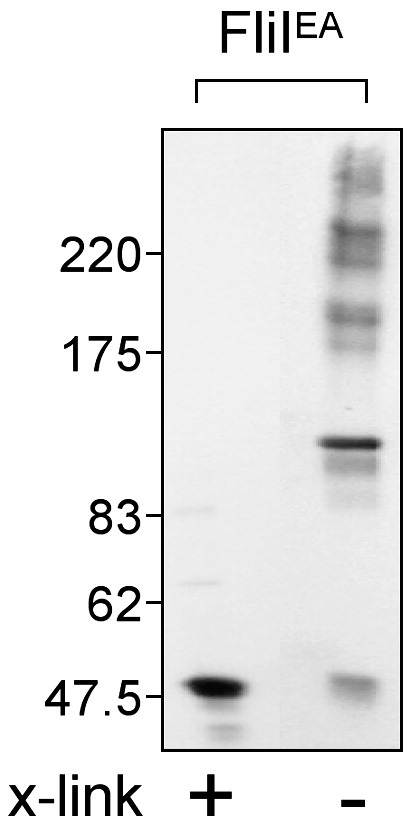
*In vitro* oligomerisation of FliI_E211A_ ATPase (FliI^EA^). Purified variant FliI ATPase (0.5 μM) was incubated in cross-linking buffer (20 mM Hepes pH 8.0, 0.1 M NaCl, 0.1 mM ethylenediaminetetraacetic acid and 1 mM DTT) in the presence of *E. coli* liposomes, with (+) or without (−) 0.1 mM disuccinimidylglutamate). Aliquots were precipitated (10% trichloroacetic acid), subjected to electrophoresis through an SDS 4–10% acrylamide gradient gel and Coomassie stained.
